# HER2 gene amplification in esophageal squamous cell carcinoma is less than in gastroesophageal junction and gastric adenocarcinoma

**DOI:** 10.3892/ol.2013.1348

**Published:** 2013-05-15

**Authors:** JUN-XING HUANG, KUN ZHAO, MEI LIN, QI WANG, WEI XIAO, MAO-SONG LIN, HONG YU, PING CHEN, RONG-YU QIAN

**Affiliations:** 1 Departments of Oncology, The People’s Hospital of Taizhou, Taizhou Medical School, Jiangsu and Nantong University, Taizhou, Jiangsu 225300, P.R. China; 2 Pathology, The People’s Hospital of Taizhou, Taizhou Medical School, Jiangsu and Nantong University, Taizhou, Jiangsu 225300, P.R. China

**Keywords:** esophageal cancer, gastric cancer, human epidermal growth factor receptor 2 gene, fluorescence *in situ* hybridization

## Abstract

The aim of the present study was to detect the amplification of the human epidermal growth factor receptor 2 (HER2) gene in esophageal squamous cell carcinoma (ESCC), gastroesophageal junction adenocarcinoma (GEJAC) and gastric cancer (GC), as well as to understand the pathological meaning of HER2 gene amplification with regard to clinico-pathological parameters in these types of cancer. HER2 gene amplification was evaluated by fluorescence *in situ* hybridization (FISH) in surgically obtained specimens from 76 cases of ESCC, 50 of GEJAC and 48 of GC, as well as 21 specimens of tumor-adjacent normal epithelium as a control group. The HER2 gene amplification rates in ESCC, GEJAC and GC were 3.9 (3/76), 24.0 (12/50) and 18.8% (9/48), respectively. The rates of HER2 gene amplification in GEJAC and GC were significantly higher compared with ESCC (χ^2^=11.563, P<0.001 and χ^2^=7.375, P<0.007, respectively). HER2 gene amplification was not detected in the normal esophageal or gastric mucosa samples. In ESCC, HER2 gene amplification was correlated with the invasion of the ESCC cells, vascular invasion and lymph node metastasis (χ^2^=4.789, 3.858 and 5.354, respectively; all P<0.05). However, in GEJAC and GC, no correlations were observed between HER2 amplification and the gender, age, degree of differentiation, invasion, vascular invasion and lymph node metastases of the patients (all P>0.05). The rate of HER-2 gene amplification was low in ESCC, although the amplification of HER-2 was correlated with tumor metastasis in these patients. The rates of HER-2 gene amplification in GEJAC and GC were higher compared with ESCC. Therefore, compared with ESCC, GEJAC may be more similar to GC with regard to HER-2 gene amplification features.

## Introduction

Esophageal cancer is a leading cause of cancer mortality worldwide and is the eighth most common cause of cancer-associated mortality (1). The incidence rate of adenocarcinoma of the esophagus has been increasing in several Western countries (2). Esophageal squamous cell carcinoma (ESCC) comprises ∼90% of esophageal cancer in China and is the fourth most common cause of mortality. Furthermore, the morbidity of gastroesophageal junction adenocarcinoma (GEJAC) has become significantly higher. In addition, there is much debate concerning the standard treatment for GEJAC, and the molecular mechanisms underlying its initiation remain poorly understood (3). Despite modest improvements in survival with either pre-operative chemotherapy or combined chemoradiotherapy in conjunction with surgery, the majority of patients with localized disease develop metastatic disease (4). Systemic chemotherapy in metastatic esophageal cancer has limited effectiveness, with responses observed in 20–40% of patients, resulting in a median survival time of 8–10 months (5). The limited effect of systemic therapy demonstrates the necessity of identifying new active agents, therapeutic strategies and therapeutic targets.

With the advent of the era of genomic science, the development of tumor-targeted drug therapy has increased rapidly. Human epidermal growth factor receptor 2 (HER2)-related signaling is reported to have an significant role in modulating cell proliferation, survival, migration and differentiation, and is therefore an effective target for molecular targeted therapy. Trastuzumab is an anti-HER2-targeting therapy that has been developed that uses humanized antibodies against HER2. Differences in HER2 dysregulation in primary solid tumors and metastases may, at least partially, explain HER2-targeted therapeutic inconsistencies. Trastuzumab has been approved for the treatment of advanced gastric cancer (GC) and GEJAC (6–9). While, HER2 is overexpressed in a number of cancers, the rate of HER2 amplification is variable in esophageal cancer (10) and few studies have investigated the different features of HER2 gene amplification among ESCC, GEJAC and GC. The aim of the present study was to simultaneously investigate the amplification status of HER2 in ESCC, GEJAC and GC tissues and use the same method to analyze its clinical significance. The present study is likely to provide more evidence for further targeted therapy in patients with esophageal cancer.

## Materials and methods

### Patients and specimens

All specimens were obtained from patients who had not received chemotherapy or radiotherapy prior to surgical resection. Patients with middle and lower ESCC, GEJAC and distal GC (76, 50 and 48 cases, respectively) underwent surgical resection at the Department of Thoracic and General Surgery of The People’s Hospital of Taizhou (Taizhou Medical School, Jiangsu and Nantong University, Taizhou, China), between August 2008 and September 2010. All patients had undergone subtotal or total esophagectomy, radical lymph node dissection and radical GC resection. This study was approved by the ethics committee of The People’s Hospital of Taizhou, Jiangsu, China.

Histopathological specimens were fixed in 10% buffered formalin, routinely processed and embedded in paraffin. All hematoxylin and eosin stained sections were reviewed and reexamined by pathologists. The grade of tumor differentiation was determined according to the classification of the World Health Organization (11) and staged according to the TNM classification (12). Normal esophageal or gastric tissue samples were obtained from 21 patients from an area >5 cm from the cancerous tissue, as control non-tumor samples.

### Fluorescence in situ hybridization (FISH)

The PathVysion HER-2 DNA Probe kit (Abbott Laboratories, Chicago, IL, USA) was used for HER2 FISH amplification; the PathVysion DNA probe kit uses a dual-color probe to determine the number of copies of HER2 (orange) and chromosome 17 centromeres (CEP-17; green). The specimen pretreatment process and degeneration of the hybrid were performed in strict accordance with the manufacturer’s instructions. Briefly, the tumor slides were deparaffinized with xylene, dehydrated in 100% ethanol at room temperature and finally air-dried in a slide warmer at 45–50°C. The slides were then pretreated by immersion in 0.2 M HCl for 20 min, purified water for 3 min, wash buffer for 3 min, pretreatment solution at 80°C for 30 min, purified water for 1 min and wash buffer for 5 min. The slides underwent protease treatment by immersion in protease solution for 10 min at 37°C and immersion in wash buffer for 5 min, followed by being air-dried on a slide warmer. Subsequently, the slides were subjected to denaturation by immersion in denaturing solution at 72±1°C for 5 min, followed by immersion in 70% ethanol for 1 min, 85% ethanol for 1 min, 100% ethanol for 1 min and then being air-dried on a slide warmer. Hybridization was then performed by applying 10 *μ*l probe mixture to the target area of the slide. Next, a glass cover slip was placed over the probe to allow even spreading and the edges of the cover slip were sealed with rubber cement. The slides were placed into a prewarmed humidified hybridization chamber, then incubated at 83°C for 5 min and at 42°C for 16 h. After removing the cover slips and rubber cement, the slides were immersed in 0.1% NP-40/0.4X SSC at 46°C for 5 min. The slides were then air-dried in the dark in an upright position. A 4′,6-diamidino-2-phenylindole (DAPI) counterstain (20 *μ*l) was applied to the target areas of the slide, which were then protected with a coverslip. The slides were stored in the dark at −20°C. For long-term preservation, neutral gum was used to seal the coverslip.

### Data analysis

Non-overlapping cells of the same nuclear size, boundary integrity, dyeing uniformity and green CEP-17 signal were selected. The double-color signals were counted randomly and a standard interpretation method was used (13). A minimum of 20 nuclei were scored by two observers using an Olympus BX 41 fluorescent microscope (Olympus Optical Co., Ltd., Tokyo, Japan) with a Chroma filter set (DAPI/spectrum orange/spectrum green triple bandpass). The areas scored were limited to regions of invasive disease according to a companion hematoxylin and eosin-stained section. The ratio of HER2 signals (orange) to CEP-17 signals (green) was calculated. The HER2 gene was considered to be amplified if there were more than six HER2 gene copies per nucleus or if there was a FISH ratio (HER2 gene to CEP-17 signals) of >2.2. HER2 was considered to be negative if the ratio was <1.8. If the ratio ranged between 1.8 and 2.2, the signal of 20 more nuclei was counted or the signal was counted again by another analyst. The FISH results were interpreted independently in a blinded manner by three pathologists.

### Statistical analysis

The χ^2^ test was used to evaluate the differences between two groups. In all tests, P<0.05 was considered to indicate a statistically significant difference. Statistical calculations were performed using SPSS 16.0 (SPSS Inc., Chicago, IL, USA).

## Results

### HER2 amplification in tumor tissue

**Figure 1.**
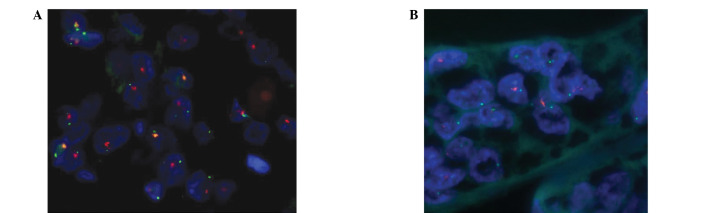
(A) Positive and (B) negative HER2 gene amplification in ESCC (FISH, magnification ×1,000). HER2, human epidermal growth factor receptor 2; ESCC, esophageal squamous cell carcinoma; FISH, fluorescence *in situ* hybridization.

**Figure 2.**
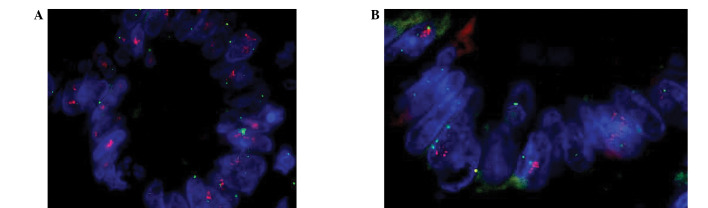
(A) Positive and (B) negative HER2 gene amplification in GC (FISH, magnification ×1,000). HER2, human epidermal growth factor receptor 2; GC, gastric cancer; FISH, fluorescence *in situ* hybridization.

**Figure 3.**
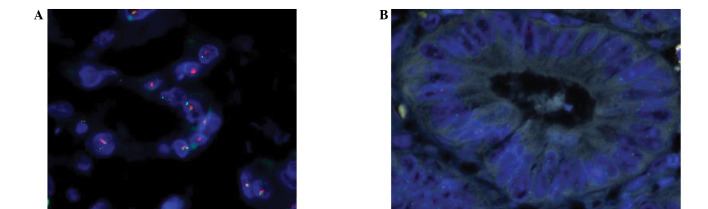
(A) Positive and (B) negative HER2 gene amplification in GEJAC (FISH, magnification ×1,000). HER2, human epidermal growth factor receptor 2; GEJAC, gastroesophageal junction adenocarcinoma; FISH, fluorescence *in situ* hybridization.

The correlations between the rate of HER2 gene amplification and ESCC, GEJAC, GC and normal esophageal or gastric mucous membrane tissues are shown in Table I. The rates of HER2 gene amplification in ESCC, GEJAC and GC were 3.9 (3/76), 24.0 (12/50) and 18.8% (9/48), respectively (Figs. 1–3). HER2 gene amplification was not identified in the normal esophageal or gastric mucosa samples. No differences were observed in HER2 gene amplification between the normal esophageal mucosa and ESCC tissues (χ^2^=0.855, P=0.355). HER2 gene amplification was significantly higher in the GEJAC and GC samples compared with the normal mucosa tissues (χ^2^=6.065, P=0.014 and χ^2^=4.528, P=0.033, respectively).

### Comparison of HER2 gene amplification and clinicopathological features

HER2 gene amplification in ESCC was markedly correlated with the tumor local infiltration, venous invasion and lymph node metastasis (χ^2^=4.789, 3.858 and 5.354, respectively; all P<0.05), but was not correlated with the gender, age and tumor differentiation of the patient. For GEJAC and GC, HER2 gene amplification was not associated with local infiltration, venous invasion, lymph node metastasis or the gender, age and tumor differentiation of the patient (P>0.05; Table II).

### Comparison of HER2 gene amplification in ESCC, GEJAC and GC

The rates of HER2 gene amplification in GEJAC and GC were significantly higher than in ESCC (χ^2^=11.563, P<0.001 and χ^2^=7.375, P<0.007, respectively; Table III). The HER2 gene amplification status in the patients with GEJAC was more similar to GC compared with ESCC (Tables II and III).

## Discussion

Overexpression of HER2 is common in multi-type tumors, such as breast and ovarian cancer. There are clear correlations between HER2 gene amplification and tumor invasiveness and metastasis, chemotherapy resistance and poor prognoses (14). Overamplification of the HER2 gene has been shown to have a significant role in tumor development. Trastuzumab, a monoclonal antibody against the HER2 receptor, has been used with success in primary and HER2-positive metastatic breast cancers. A phase III ToGA trial designed to assess the effect of trastuzumab in patients with HER2-positive GCs reported that the addition of trastuzumab to chemotherapy significantly improved overall survival without compromising safety in patients with HER2-positive metastatic gastric or gastroesophageal junction cancer (15). Therefore, it is important to thoroughly investigate the HER2 amplification status in gastric and esophageal carcinomas (16).

There is controversy with regard to the status of HER2 expression in ESCC. The rate of HER2 gene amplification has been recorded as between 6.5 and 7.5% in certain studies (17,18), while the results of the majority of studies were within 30%. Wu *et al* reported that the rate of HER2 over-expression was 14.1% according to immunohistochemistry (IHC) in ESCC (19), while HER2 positivity was demonstrated in 17% of resected esophageal adenocarcinomas in a study by Yoon *et al* (20) and HER2 protein overexpression was 10.4% in ESCC according to the study by Zhan *et al* (21). The present study showed that the rate of HER2 gene amplification was 3.9% in patients with ESCC. The reasons for this diversity in the amplification rates of the present study and the literature are unclear. One of the reasons may be that the present study cases were all ESCC and the method of study used was FISH. Another explanation may be that there is wide geographical variation in the incidence of ESCC in the world, indicating that ESCC has heterogeneity and diversity in its molecular and clinical manifestation. The methods used to study HER2 gene amplification in patients with ESCC have included IHC and FISH (22). Detecting HER2 gene amplification with FISH may increase accuracy and make the use of anti-HER2 targeted therapies more precise. A number of systematic reviews have considered FISH to be more objective and reproducible (23), so it is regarded as the gold standard for HER2 gene detection.

Similarly, there have been various conclusions concerning the association between HER2 gene amplification and clinicopathological characteristics in patients with ESCC. One study suggested that HER2 overexpression was not significantly associated with the clinicopathological characteristics of patients with ESCC (19). Zhan *et al* reported that there were correlations between the overexpression of HER2 and the differentiation of the carcinoma, HER2 gene amplification and the differentiation of the carcinoma and tumor stage (21). By contrast, HER2 positivity was associated with reduced tumor aggressiveness and independently associated with improved survival in resected esophageal adenocarcinoma, according to a study at the Mayo Clinic (20). The present results showed that low HER2 amplification only occurred in patients with ESCC and that it was associated with tumor infiltration depth and vascular and lymph node metastases. Thus, HER2 expression status and its significance in esophageal cancer should be investigated further.

The present results showed that the rate of HER2 gene amplification was 18.8% in patients with GC. The reported rates of HER2 gene amplification are also different in the literature for GC. There is a general consensus that the overexpression of this receptor occurs in ∼20% of gastric adenocarcinomas (24). Several groups have reported that the rate of HER2 gene amplification was between 9.0 and 15.9% in GC (25–27). Barros-Silva observed that HER2 amplification was not notably correlated with gender, age, vascular tumor thrombus, lymph node metastasis or clinical staging in patients with GC (28). This result agreed with that of the present study.

At present, there is disagreement concerning the common definition of GEJAC (29,30). It has been reported, that the rate of HER2 gene amplification is 16–32% in GEJAC, which is higher than in GC (31–34). Grávalos *et al* reported that the HER2-positive rate was 10% in advanced gastric and gastroesophageal junction adenocarcinoma (35). In the present study, the rate of HER2 gene amplification in GEJAC (24.0%) was slightly higher than in GC (18.8%), although the difference was not statistically significant. Furthermore, the rate of gene amplification was significantly different between the patients with ESCC (3.9%) and the patients with GEJC and GC. By comparing the clinicopathological features of three types of tumor, the features of HER-2 amplification in GEJAC were observed to be more similar to GC. This result may contribute to the further definition of GEJAC and the differential treatment strategies for various subtypes in patients with GEJAC.

Taken together, the present data indicated that HER2 amplification was higher in GEJAC and GC, but lower in ESCC. Targeting HER2 may be a suitable choice for patients with GC and GEJAC, while targeting HER2 in ESCC requires further study.

## Figures and Tables

**Table I.** HER2 gene amplification in ESCC, GEJAC and GC and in normal epithelial specimens.

Group	n	Positive amplification	Positive rate (%)	χ^2^	P-value
Normal specimens	21	0	0.0	-	-
ESCC	76	3	3.9	0.855	0.355
GEJAC	50	12	24.0	6.065	0.014
GC	48	9	18.8	4.528	0.033

HER2, human epidermal growth factor receptor 2; ESCC, esophageal squamous cell carcinoma; GEJAC, gastroesophageal junction adenocarcinoma; GC, gastric cancer.

**Table II.** Correlations between HER2 gene amplification and clinicopathological parameters.

Characteristic	ESCC				GEJAC				GC			
	n	HER2^+^	χ^2^	P value	n	HER2^+^	χ^2^	P value	n	HER2^+^	χ^2^	P value
Gender												
Male	57	3			38	8			41	6		
Female	19	0	1.041	0.308	12	4	0.754	0.385	17	3	0.083	0.773
Age												
<65	46	2			24	4			33	7		
≥65	30	1	0.049	0.824	26	8	1.361	0.243	25	2	1.894	0.169
Differentiation												
Well, moderately	56	2			24	7			18	4		
Poorly	20	1	0.079	0.778	26	5	0.675	0.411	40	5	0.895	0.344
Depth of invasion												
T1+T2	46	0			18	6			22	4		
T3+T4	30	3	4.789	0.029	32	6	1.343	0.246	36	5	0.192	0.661
Vascular invasion												
Yes	34	3			40	8			43	5		
No	42	0	3.858	0.050	10	4	1.754	0.185	15	4	1.919	0.166
Lymph node metastasis												
Yes	28	3			28	7			31	4		
No	48	0	5.354	0.021	22	5	0.035	0.852	27	5	0.347	0.556

HER2, human epidermal growth factor receptor 2; ESCC, esophageal squamous cell carcinoma; GEJAC, gastroesophageal junction adenocarcinoma; GC, gastric cancer.

**Table III.** Correlations with HER2 gene amplification in ESCC, GEJAC and GC.

	n	Positive amplification	χ^2^	P-value
ESCC/GEJAC	76/50	3/12	11.563	0.001
ESCC/GC	76/48	3/9	7.375	0.007
GEJAC/GC	50/48	12/9	0.401	0.527

HER2, human epidermal growth factor receptor 2; ESCC, esophageal squamous cell carcinoma; GEJAC, gastroesophageal junction adenocarcinoma; GC, gastric cancer.

## References

[1] Kamangar F, Dores GM, Anderson WF (2006). Patterns of cancer incidence, mortality, and prevalence across five continents: defining priorities to reduce cancer disparities in different geographic regions of the world. J Clin Oncol.

[2] Jemal A, Bray F, Center MM, Ferlay J, Ward E, Forman D (2011). Global cancer statistics. CA Cancer J Clin.

[3] Liakakos T, Katsios C, Roukos DH (2011). Gastroesophageal junction carcinoma multimodal treatment: standards, debate and new therapeutic options. Expert Rev Gastroenterol Hepatol.

[4] Ilson DH (2008). Esophageal cancer chemotherapy: recent advances. Gastrointest Cancer Res.

[5] Ku GY, Ilson DH (2007). Esophageal cancer: adjuvant therapy. Cancer J.

[6] Okines AF, Cunningham D (2012). Trastuzumab: a novel standard option for patients with HER-2-positive advanced gastric or gastro-oesophageal junction cancer. Therap Adv Gastroenterol.

[7] Dai GH, Shi Y, Chen L, Lv YL, Zhong M (2012). Trastuzumab combined with docetaxel-based regimens in previously treated metastatic gastric cancer patients with HER2 over-expression. Hepatogastroenterology.

[8] Hicks DG, Whitney-Miller C (2011). HER2 testing in gastric and gastroesophageal junction cancers: a new therapeutic target and diagnostic challenge. Appl Immunohistochem Mol Morphol.

[9] Lordick F (2011). Trastuzumab: a new treatment option for HER2-positive metastatic gastric and gastroesophageal junction cancer. Future Oncol.

[10] Reichelt U, Duesedau P, Tsourlakis MCh (2007). Frequent homogeneous HER-2 amplification in primary and metastatic adenocarcinoma of the esophagus. Mod Pathol.

[11] Gabbert HE, Nakamura Y, Shimoda T, Field JK, Hainaut P, Inoue H, Hamilton SR, Aaltonen LA (2000). Squamous cell carcinoma of the oesophagus. World Health Organization Classification of Tumors.

[12] Huang J, Sun Y, Shi YK (2007). Esophageal cancer. Manual of Medical Oncology.

[13] Wolff AC, Hammond ME, Schwartz JN (2007). American Society of Clinical Oncology/College of American Pathologists guideline recommendations for human epidermal growth factor receptor 2 testing in breast cancer. J Clin Oncol.

[14] Dent R, Trudeau M, Pritchard KI (2007). Triple-negative breast cancer: clinical features and patterns of recurrence. Clin Cancer Res.

[15] Bang YJ, Van Cutsem E, Feyereislova A (2010). Trastuzumab in combination with chemotherapy versus chemotherapy alone for treatment of HER2-positive advanced gastric or gastro-oesophageal junction cancer (ToGA): a phase 3, open-label, randomised controlled trial. Lancet.

[16] Koltz BR, Hicks DG, Whitney-Miller CL (2012). HER2 testing in gastric and esophageal adenocarcinoma: new diagnostic challenges arising from new therapeutic options. Biotech Histochem.

[17] Sato-Kuwabara Y, Neves JI, Fregnani JH, Sallum RA, Soares FA (2009). Evaluation of gene amplification and protein expression of HER-2/neu in esophageal squamous cell carcinoma using Fluorescence in situ Hybridization (FISH) and immunohistochemistry. BMC Cancer.

[18] Wei Q, Chen L, Sheng L, Nordgren H, Wester K, Carlsson J (2007). EGFR, HER2 and HER3 expression in esophageal primary tumours and corresponding metastases. Int J Oncol.

[19] Wu D, Xu J, Yu G (2012). Expression status of fatty acid synthase (FAS) but not HER2 is correlated with the differentiation grade and prognosis of esophageal carcinoma. Hepatogastroenterology.

[20] Yoon HH, Shi Q, Sukov WR (2012). Association of HER2/ErbB2 expression and gene amplification with pathologic features and prognosis in esophageal adenocarcinomas. Clin Cancer Res.

[21] Zhan N, Dong WG, Tang YF, Wang ZS, Xiong CL (2012). Analysis of HER2 gene amplification and protein expression in esophageal squamous cell carcinoma. Med Oncol.

[22] Varshney D, Zhou YY, Geller SA, Alsabeh R (2004). Determination of HER-2 status and chromosome 17 polysomy in breast carcinomas by comparing HercepTest and PathVysion FISH assay. Am J Clin Pathol.

[23] Ross JS (2011). Point: Fluorescence *in situ* hybridization is the preferred approach over immunohistochemistry for determining HER2 status. Clin Chem.

[24] Pazo Cid RA, Antón A (2013). Advanced HER2-positive gastric cancer: Current and future targeted therapies. Crit Rev Oncol Hematol.

[25] Terashima M, Kitada K, Ochiai A (2012). Impact of expression of human epidermal growth factor receptors EGFR and ERBB2 on survival in stage II/III gastric cancer. Clin Cancer Res.

[26] Shitara K, Yatabe Y, Matsuo K (2012). Prognosis of patients with advanced gastric cancer by HER2 status and trastuzumab treatment. Gastric Cancer.

[27] Kim JW, Im SA, Kim M (2012). The prognostic significance of HER2 positivity for advanced gastric cancer patients undergoing first-line modified FOLFOX-6 regimen. Anticancer Res.

[28] Barros-Silva JD, Leitão D, Afonso L (2009). Association of ERBB2 gene status with histopathological parameters and disease specific survival in gastric carcinoma patients. Br J Cancer.

[29] Rüschoff J (2012). Adenocarcinoma of the GEJ: gastric or oesophageal cancer?. Recent Results Cancer Res.

[30] Suh YS, Han DS, Kong SH (2012). Should adenocarcinoma of the esophagogastric junction be classified as esophageal cancer? A comparative analysis according to the seventh AJCC TNM classification. Ann Surg.

[31] Lorenzen S, Lordick F (2011). How will human epidermal growth factor receptor 2-neu data impact clinical management of gastric cancer?. Curr Opin Oncol.

[32] Fassan M, Ludwig K, Pizzi M (2012). Human epithelial growth factor receptor 2 (HER2) status in primary and metastatic esophagogastric junction adenocarcinomas. Hum Pathol.

[33] Wainberg ZA, Lin LS, DiCarlo B (2011). Phase II trial of modified FOLFOX6 and erlotinib in patients with metastatic or advanced adenocarcinoma of the oesophagus and gastro-oesophageal junction. Br J Cancer.

[34] Thompson SK, Sullivan TR, Davies R, Ruszkiewicz AR (2011). Her-2/neu gene amplification in esophageal adenocarcinoma and its influence on survival. Ann Surg Oncol.

[35] Grávalos C, Gómez-Martín C, Rivera F, Alés I (2011). Phase II study of trastuzumab and cisplatin as first-line therapy in patients with HER2-positive advanced gastric or gastroesophageal junction cancer. Clin Transl Oncol.

